# Protective Effect and Potential Mechanism of the Traditional Chinese Medicine Shaoyao-Gancao Decoction on Ethanol-Induced Gastric Ulcers in Rats

**DOI:** 10.1155/2022/3069089

**Published:** 2022-04-11

**Authors:** Yang Jin, Ming Zhang, Yuefei Wang, Yuan Lu, Ting Liu, Guiqian Yang, Shuoyao Song, Wen Liu

**Affiliations:** ^1^School of Basic Medical Sciences, Guizhou Medical University, Guiyang 550004, China; ^2^The Key Laboratory of Chemistry for Natural Products of Guizhou Province, Chinese Academy of Sciences, Guiyang 550014, China; ^3^The First Affiliated Hospital of Guizhou University of Traditional Chinese Medicine, Guiyang 550001, China; ^4^Provincial Key Laboratory of Pharmaceutics in Guizhou Province, Guizhou Medical University, Guiyang 550004, China; ^5^School of Pharmaceutical Sciences, Guizhou Medical University, Guiyang 550004, China; ^6^Affiliated Hospital of Guizhou Medical University, Guiyang 550004, China

## Abstract

**Background:**

Shaoyao-Gancao decoction (SGD) is a classic prescription in traditional Chinese medicine. SGD is effective in the treatment of gastric and duodenal ulcers. However, the biological activity and possible mechanisms of SGD in the treatment of gastric ulcers have not been fully elucidated. The purpose of this study was to scientifically evaluate the protective effect and potential mechanism of SGD against ethanol-induced gastric ulcers in rats.

**Methods:**

A single gavage of 10 mL/kg of 75% ethanol was used to establish a rat gastric ulcer model. A histopathological examination of the gastric tissue was performed. The levels of TNF-*α*, EGF, PGE_2_, SOD, and TBARS in gastric tissue were measured by ELISA. Cellular apoptosis in gastric tissues was assessed by TUNEL assay. The expression levels of caspase-3 and Bcl-2 were determined by immunohistochemistry. The potential mechanism of SGD in treating gastric ulcers was further studied using a network pharmacology research method.

**Results:**

The gastric tissue of rats with ethanol-induced gastric ulcers had obvious injury throughout the mucosal layer, which was significantly weakened in rats treated with SGD. Furthermore, treatment with SGD significantly increased the levels of EGF, PGE_2_, SOD, and Bcl-2 and decreased the levels of TNF-*α*, TBARS, and caspase-3 in the gastric tissue of rats with ethanol-induced gastric ulcers. SGD reduced ethanol-induced cell apoptosis in gastric tissue from rats with gastric ulcers. A traditional Chinese medicine-based network pharmacology study revealed that SGD exerts its anti-gastric ulcer effect by acting on multiple pathways.

**Conclusions:**

The above results indicate that SGD can improve gastric ulcers induced by ethanol. Moreover, this study demonstrated multicomponent, multitarget, and multipathway characteristics of SGD in the treatment of gastric ulcers and provided a foundation for further drug development research.

## 1. Introduction

Gastric ulcer (GU) is one of the most common diseases affecting the digestive system, and it affects millions of people around the world. GU has three important clinical manifestations, namely a high incidence, a high recurrence rate, and a high canceration rate. It has been listed by the World Health Organization (WHO) as one of the main precancerous lesions and is a serious threat to human life and health [1–3]. Symptoms of GU may include one or more of the following: bloating, abdominal pain, loss of appetite, weight loss, nausea, and vomiting. The pathogenesis of GU is complicated, and although its exact pathophysiological mechanism is not completely clear, it is well known that GU is mainly caused by the imbalance of gastric invasiveness and defensive factors [4, 5]. The disease can be caused by a variety of endogenous and exogenous causes, such as *Helicobacter pylori* infection, gastric hyperchloremia, gastric mucosal ischemia, poor diet, stress, smoking, long-term use of nonsteroidal anti-inflammatory drugs (NSAIDs), and excessive alcohol consumption [6–9]. The conventional drugs used in the treatment of GU include proton pump inhibitors, antacids, H2 receptor antagonists, and anticholinergic drugs [10]. However, the efficacy of current treatments is not absolute, and they may have serious side effects, such as gastrointestinal reactions, rebound effects, hypergastrinemia, hepatorenal toxicity, allergies, and arrhythmias [11–16]. Therefore, many scholars have begun to look for natural products or natural resources with therapeutic activity against GU to replace drugs with strong side effects [17–19]. Botanical drugs have attracted increasing attention because of their efficacy, safety, relatively low cost, and compatibility with the human system. Many studies have proven the gastroprotective effects of plant extracts and compound prescriptions from traditional Chinese medicine, such as *Bletilla striata* polysaccharide, safranal, patchoulene epoxide, and Lizhong decoction [20–23].

Shaoyao-Gancao decoction (SGD) is a classic prescription from traditional Chinese medicine that has been used since it was first recorded in the Treatise on Febrile Diseases written by Zhang Zhongjing in the Han Dynasty. SGD is composed of *Paeonia lactiflora Pall* (TCM herb name Bai Shao (BS)) and *Glycyrrhiza uralensis Fisch* (TCM herb name Zhi Gan Cao (ZGC)) at a ratio of 1 : 1. SGD made by combining these two herbs has two-way regulatory, antispasmodic, analgesic, and other pharmacological effects and has good curative effects on various diseases. Clinically, it is mainly used to treat diseases characterized by visceral smooth muscle colic and severe cramps, such as GU and duodenal ulcers. SGD has a good curative effect and warrants further research. Hence, the gastroprotective effect of SGD on GU and the underlying mechanism deserves comprehensive elucidation.

**Figure 1:**
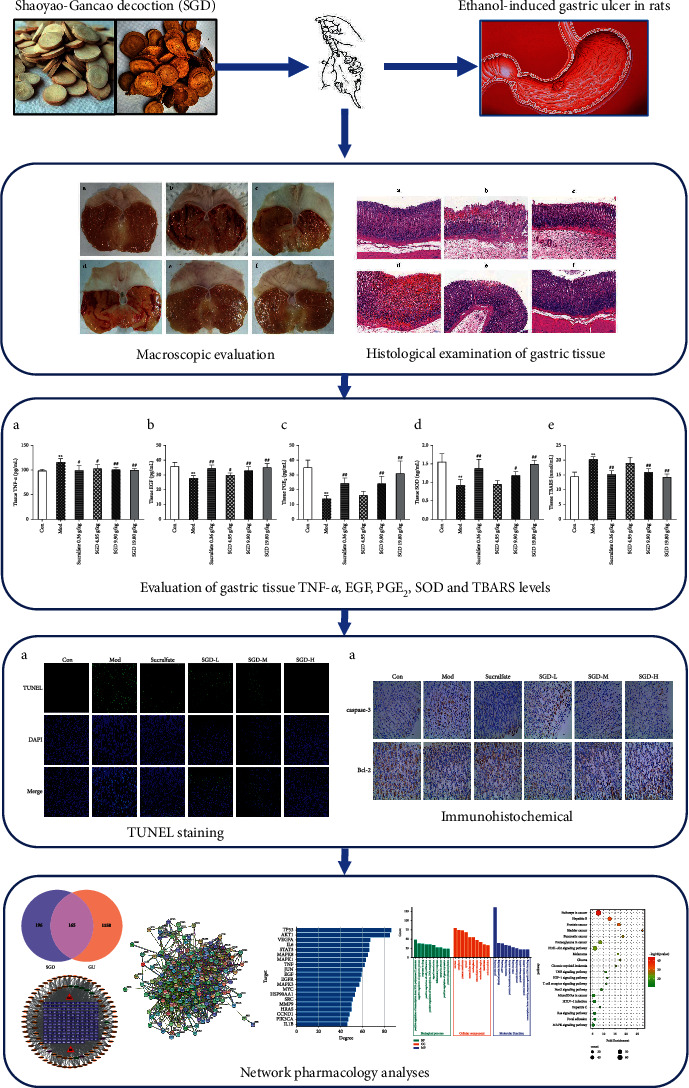
Workflow chart of this research.

Modern pharmacological studies have shown that a high concentration of ethanol can erode the gastric mucosa within 30–60 min, resulting in upper gastrointestinal bleeding and gastric mucosal lesions [24, 25]. Therefore, ethanol is often used to test the gastroprotective properties of drugs due to its damage to the gastric mucosa. In addition, the ethanol-induced GU model is more similar to human acute peptic ulcers and is superior to other models (such as stress, pyloric ligation, and NSAID induction) [26] and independent of gastric acid secretions. Depending on the type of animal model used, positive controls such as sucralfate and misoprostol are used for cytoprotective effects, and drugs such as H2 receptor blockers and proton pump inhibitors are used for antisecretory effects [27–30]. According to previous reports, people who drink alcohol have a higher incidence of GU-related conditions such as gastritis, GU, and gastric cancer [31, 32]. The pathology of ethanol-induced GU generally involves three dimensions: oxidative stress, apoptosis, and the inflammatory response. Thus, in this study, the gastroprotective effects of SGD against ethanol-induced GU in rats were investigated with macroscopic and histopathological evaluation systems. Inflammatory cytokine release, production of protective mediators in the gastric mucosa, oxidative stress status, and cell apoptosis were further investigated. Finally, using the network pharmacology research method, we constructed the SGD component-target-disease network and further studied the potential mechanism underlying the anti-GU effect of SGD through enrichment analysis of GO functions and KEGG pathways. The flow diagram of the study is shown in Figure 1.

## 2. Materials and Methods

### 2.1. Chemicals and Reagents

Sucralfate oral suspension (Lot No. KF201003) was purchased from Shanghai Xudong Haipu Pharmaceutical Co., Ltd. (Shanghai, China). Absolute ethanol solution (Lot No. GB678-90) was acquired from Tianjin Xinbote Chemical Co., Ltd. (Tianjin, China). Xylene (Lot No. 20200601) was purchased from Tianjin Zhiyuan Chemical Reagent Co., Ltd. (Tianjin, China). Neutral formalin fixative (10%; Lot No. DF0111) and DAPI staining solution (5 *μ*g/mL, Lot No. DA0001) were obtained from Beijing Legian Biotechnology Co., Ltd. (Beijing, China). Hematoxylin stain (Lot No. ZH193907) was acquired from Wuhan Seville Biotechnology Co., Ltd. (Wuhan, China). Eosin stain (Lot No. C200403) was purchased from Zhuhai Baso Biotechnology Co., Ltd. (Zhuhai, China), and PBS powder (Lot No. ZLI-9062), goat anti-rabbit/mouse working solution (Lot No. SP-9001/2), normal goat serum (Lot No. ZLI-9021), and concentrated DAB kits (Lot No. K135925C) were obtained from Beijing Zhongshan Jinqiao Biotechnology Co., Ltd. (Beijing, China). The caspase-3 mouse monoclonal antibody (Lot No. 66470-2-lg) was acquired from ProteinTech. The Bcl-2 rabbit polyclonal antibody (Lot No. bs-0279R) was purchased from Beijing Boaosen Biotechnology Co., Ltd. (Beijing, China). A TUNEL apoptosis kit (Lot No. 11684795910) was obtained from Roche Group. Kits for the determination of tumor necrosis factor-*α* (TNF-*α*, Cat.# ZC-37624, cutoff value: 1.0 pg/mL), superoxide dismutase (SOD, Cat.# ZC-36451, cutoff value: 0.1 ng/mL), thiobarbituric acid reactive substances (TBARS, Cat.# ZC-36981, cutoff value: 0.1 nmol/mL), prostaglandin E_2_ (PGE_2_, Cat.# ZC-37100, cutoff value: 1.0 pg/mL), and epidermal growth factor (EGF, Cat.# ZC-36425, cutoff value: 1.0 pg/mL) were acquired from Shanghai Zhuokai Biotechnology Co., Ltd. (Shanghai, China). All chemicals used in buffers and other solutions were of analytical grade and obtained from regular commercial suppliers.

### 2.2. Sources and Authentication of Herbs

BS (Lot No. 20020101, origin Anhui) and ZGC (Lot No. 200316, origin Gansu) were purchased from Sinopharm Tongjitang (Guizhou) Pharmaceutical Co., Ltd. (Guiyang, China). Based on the Chinese Pharmacopoeia (Version 2020), herbal medicines were authenticated by Qing-De Long of Guizhou Medical University, and voucher specimens with accession numbers 20201101 and 20201102 were deposited at Guizhou Medical University.

### 2.3. Preparation of SGD

SGD is composed of BS and ZGC at a ratio of 1 : 1. Two herbs (110 g) were immersed in cold water at a ratio of 1 : 8 (W/V, 800 mL per 100 g mixture) for 0.5 h. After boiling under strong heat, the herbs were heated under slow heat for 1 h and then filtered. The residue was further boiled with 8 volumes of cold water for 1 h and filtered again. The filtrates were combined and concentrated. The concentrate was dried under reduced pressure at 55°C. The dry extract powder for SGD was obtained, and the extracted amount was calculated. The yield of dried powder was 27.12% according to the original dry materials. An appropriate amount of ultrapure water was added and sonicated into a suspension at a final concentration of 1 g/mL before use. The content determination results of the five components of SGD are described in the supplementary materials.

### 2.4. Ethical Use of Laboratory Animals

SPF-grade male SD rats (6–8 weeks old), with a body mass of 220 ± 20 g, were maintained at the Animal Experiment Center of Guizhou Medical University (Guizhou, China; animal certificate number: SYXK (Qian) 2018-0001). All the required animals in this study were maintained in the SPF Laboratory Animal Center of Guizhou Medical University and reared at 25 ± 2°C and 40%–60% relative humidity. All rats were housed in a well-ventilated room under a 12 h light/dark cycle and had access to feed and water ad libitum. Prior to experiments, the rats were fasted for 12 h and deprived of water for 3 h. All animal experiments conformed to the Guide for the Care and Use of Laboratory Animals published by Guizhou Medical University and were approved by the Committee for Experimental Animal Ethics of Guizhou Medical University (No. 2000964). Efforts were taken to minimize animal suffering throughout the experiments.

### 2.5. Induction of GU and SGD Treatment

After one week of adaptive feeding, all the rats were randomly divided into 6 groups (*n* = 8) as follows: control group (Con), model group (Mod), positive control group (sucralfate), and SGD groups (4.95, 9.90, and 19.80 g/kg). The doses of SGD were calculated according to the raw material, and SGD was given to animals according to the human dose with human-to-animal dose conversion formula. The rats were intragastrically administered the drug once at 9 a.m. The animal equivalent dosage was determined from the human equivalent dose. During 7 days of pretreatment, rats in the Con and Mod groups were orally administered control saline (10 mL/kg), and rats in the sucralfate group were intragastrically administered 0.36 g/kg saline. The SGD groups were intragastrically administered 4.95, 9.9, and 19.8 g/kg once daily. After the administration on the sixth day, the rats were fasted for 12 h, and water was provided at will. Before administration on the seventh day, the rats were deprived of water for 3 h, and the rats were administered the treatment in the manner described above. All rats except those in the control group were given 10 mL/kg of 75% ethanol 1 h after the last administration of drugs [33–35], and the rats in the Con group were given 10 mL/kg control saline by gastric gavage. One hour after the administration of 75% ethanol, the rats were killed by cervical dislocation under anesthesia; the gastric was removed and opened along the greater curvature. The tissues were rinsed thoroughly in ice-cold physiological saline, dried using filter paper, and photographed. The morphological features of the tissue were observed to analyze tissue injury. Some gastric tissues were fixed in 10% neutralized formalin for histological, cell apoptosis, and immunohistochemistry analysis. The remaining tissues were quickly stored in a freezer at -80°C for later use.

### 2.6. Macroscopic Evaluation

The areas of ulcerated lesions were determined using ImageJ analysis software (ImageJ, 1.80, NIH, USA), and the percentages of the ulcerated areas relative to the total gastric area were calculated, namely the ulcer index (UI) [36]. The inhibition rate was calculated by the following formula:

(1) $$\begin{array}{c} \text{Inhibition}=\left[\frac{\left(\text{UI of Mod group}-\text{UI of pretreated group}\right)}{\text{UI of Mod group}}\right]\times 100\%. \end{array}$$

### 2.7. Histological Examination of Gastric Tissue

Fixed tissue was dehydrated and embedded in paraffin, cut into slices, dewaxed, and then stained using hematoxylin-eosin for microscopic examination. The images were observed and photographed with a PANNORAMIC 250 Digital Slice Scanner, and specific lesions were observed.

### 2.8. Evaluation of Gastric Tissue TNF-*α*, EGF, PGE_2_, SOD, and TBARS Levels

The gastric tissue was removed from the freezer (−80°C) and thawed. Under ice bath conditions, the pieces were cut, 9 volumes of precooled PBS solution were added, and the pieces were homogenized using an electric tissue homogenizer. The homogenates were centrifuged at 5000 rpm for 10 min at 4°C. After centrifugation, the supernatant was collected for measurement. All steps were performed strictly in accordance with the kit instructions. Finally, a multifunctional microplate reader was used to measure the absorbance of each well at a specific wavelength. TNF-*α*, EGF, PGE_2_, SOD, and TBARS levels were determined from a standard curve.

### 2.9. TUNEL Staining of Gastric Tissue

DNA fragmentation is a representative feature of late apoptotic cells, and a TUNEL apoptosis detection kit was used to detect tissue breakage via nuclear DNA during apoptosis. Gastric tissue was fixed in 10% neutral buffered formalin and processed routinely for paraffin-embedded sections. TUNEL staining assays were performed with sections using a TUNEL kit (11684795910, Roche, Switzerland) principally according to the supplier's instructions and observed under a fluorescence microscope. Nuclei were stained with DAPI. The sections were scanned by a PANNORAMIC 250 Digital Slide Scanner. TUNEL-positive nuclei within stomach tissue were counted in nine random fields for each slide with at least eight rats in each group. The percentage of apoptotic cells was calculated as (number of apoptotic cells/total number of cells) ×100.

### 2.10. Immunohistochemical Evaluation of Caspase-3 and Bcl-2

Paraffin-embedded gastric samples were sectioned, dewaxed, and dehydrated. The paraffin-embedded sections were dewaxed, placed into a staining jar, treated with 3% methanol/hydrogen peroxide at room temperature for 10 min, rinsed in PBS, and immersed in 0.01 M citrate buffer (pH 6.0). In the microwave oven, the power was cut off after the sample was heated to boiling. After an interval of 5 min, the process was repeated once.

After cooling, the sections were rinsed in PBS. The goat serum blocking solution was incubated at room temperature for 20 min, and then, the sections were incubated overnight at 4°C with a caspase-3 antibody (1 : 150) or Bcl-2 antibody (1 : 200), followed by incubation with a biotinylated secondary antibody. The secondary antibody was added dropwise, incubated at 37°C for 30 min, and then washed with PBS three times for 5 min each time. Color development was induced by incubation with a DAB kit (Dako), and the sections were counterstained with hematoxylin. Finally, the slices were dehydrated, transparentized, sealed, and mirror inspected. The images of the slices were collected by a BA200 Digital trinocular camera microcamera system. The optical density (IOD) and area (area) of all the images were measured with the Image-Pro Plus 6.0 image analysis system, the average optical density was measured, and the expression of each target protein was analyzed.

### 2.11. Network Pharmacology Analyses

#### 2.11.1. Database Construction

All compounds contained in BS and GC were searched from the Traditional Chinese Medicine Systems Pharmacology Database and Analysis Platform (TCMSP, http://tcmspw.com). Oral bioavailability (OB) refers to the speed and degree of absorption of the active compounds or active groups of the drug in the systemic circulation and is a key parameter for evaluating whether the drug can be developed. Drug likeness (DL) refers to the structural similarity between herbal ingredients and known drugs. These two parameters are the key parameters of the traditional Chinese medicine component of absorption, distribution, metabolism, and excretion (ADME). The active compounds of SGD were screened using OB ≥ 30% and DL ≥ 0.18 as the screening conditions.

#### 2.11.2. Screening of the Active Ingredients in SGD

The validated target proteins of the active components were obtained from the TCMSP database. For the components whose relevant targets were not found in TCMSP, SMILE numbers were obtained through the PubChem database (https://pubchem.ncbi.nlm. nih.gov/) and imported into the Swiss Target Prediction database (http://www.swisstargetprediction.ch/index.php) for target prediction. The target proteins with probability > 0 in the predicted targets were screened out, then the two are integrated, and the duplication is removed. The names of identified targets were normalized according to the UniProt database (https://www.uniprot.org/) with the selected species as “Homo sapiens,” and the gene name and number of the proteins were obtained. “Gastric ulcer” was used as a keyword to search the DrugBank (https://go.drugbank.com/), GeneCards (https://www.genecards.org/), OMIM (https://www.omim.org/), and DisGeNET (https://www.disgenet.org/) databases. The search results from each database were combined, duplicates were removed, and all genes and targets were submitted to the UniProt database for validation of their gene names.

#### 2.11.3. Network Establishment and Analysis

The obtained drug-related targets and the disease-related targets overlapped, and a Venn diagram (http://bioinformatics.psb.ugent.be/webtools/Venn/) of the intersected gene symbols was obtained, obtaining common targets, namely key SGD targets for the treatment of GU. The protein-protein interactions (PPIs) were analyzed with STRING version 11.0 (https://string-db.org/cgi/input.pl). In the search of the STRING database, the species was limited to “Homo sapiens” with a confidence score ≥0.7, and the PPI data were obtained. The active ingredients of SGD, the targets corresponding to the active ingredients, and the targets predicted for GU disease were imported into Cytoscape 3.7.2 software, and a drug-target-disease network diagram was constructed for network visualization. Using the online Database for Annotation, Visualization and Integrated Discovery (DAVID 6.8), the target genes in the key network modules were analyzed by determining the related GO biological processes and signaling pathways that were enriched in the KEGG. The identifier was “OFFICIAL GENE SYMBOL,” and the species was “Homo sapiens.” GO functional enrichment analysis and KEGG pathway enrichment analysis screening conditions were *P* value <0.01, and the filtered results were sorted by count value. Mapping was performed using bioinformatics software (http://www.bioinformatics.com.cn/).

### 2.12. Statistical Analysis

Statistical analysis was performed using SPSS 25.0 software, and all data are expressed as the mean ± standard deviation (SD). One-way analysis of variance was used to compare the differences among the groups. Multiple comparisons were analyzed by the least significant difference (LSD) method if the data were homogeneous. Otherwise, they were analyzed by Dunnett's T3 method. The test parameters (*α* = 0.05 and *P* value < 0.05) were considered statistically significant, and *P* < 0.01 indicated a highly significant difference. The Kruskal–Wallis analysis of variance and rank-based Mann–Whitney *U* test were used to determine the statistical significance of the ulcer index (*P* < 0.05). All data graphs were generated by GraphPad Prism 7 software (GraphPad Software, Inc., La Jolla, CA, USA).

## 3. Results

### 3.1. Macroscopic Gastric Damage

**Figure 2:**
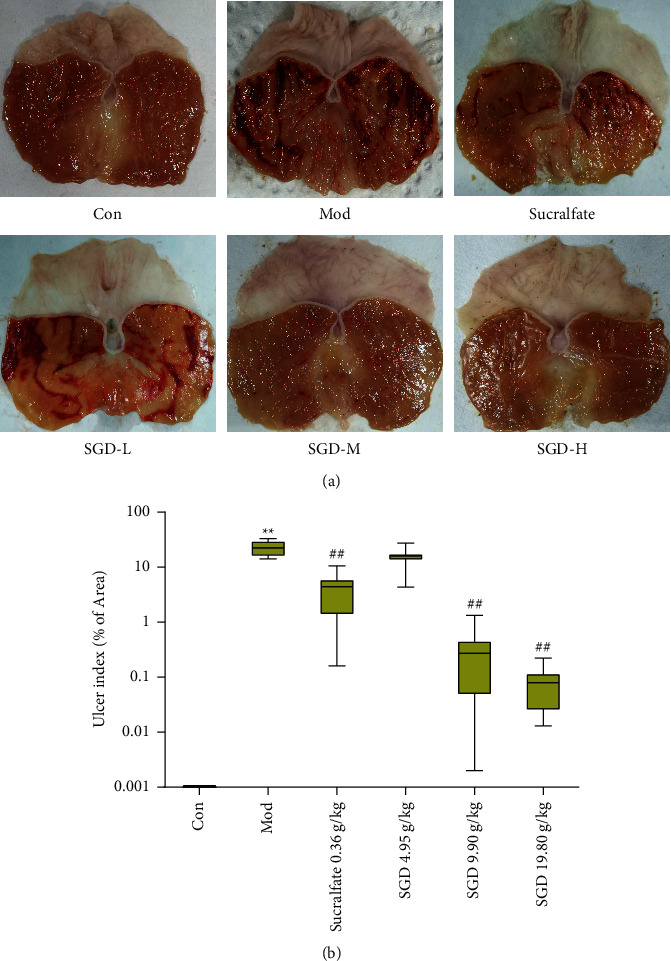
Effect of SGD on the severity of gastric mucosal lesions in model rats (*n* = 8). (a) Representative macrographs of each group. (b) Gastric ulcer index of different experimental groups. Each bar of the ulcer index represents the median (nonparametric data) for 8 rats (with interquartile range). Compared with the control group, ^*∗*^*P* < 0.05, ^*∗∗*^*P* < 0.01; compared with the model group, the remaining groups ^#^*P* < 0.05, ^##^*P* < 0.01.

As shown in Figure 2, no hyperemia, edema, erosion, or ulcer formation was detected in the gastric mucosa of rats in the control group, and the gastric mucosal surface was intact and smooth without damage. After intragastric administration of 75% ethanol for modeling, compared with that of the control group, the gastric mucosa of the model group was severely damaged, with obvious edema and congestion and dark red, striped areas of hemorrhage. Compared with the model group, the gastric mucosal injury of the model rats in the administration group showed different degrees of improvement. In the sucralfate group, the gastric mucosal hemorrhagic injury was significantly reduced, and the bleeding streaks became narrower. The rats in the SGD (4.95 g/kg) group had less gastric mucosal damage than the rats in the model group, but there was still more severe streak bleeding. The gastric mucosal injury of rats in the SGD (9.90 g/kg) group and SGD (19.80 g/kg) group was further improved; there were no striped bleeding and only short and thin linear bleeding.  Figure 2(b) shows that the gastric tissue of the rats in the control group was the control, and the ulcer index of the rats treated with 75% ethanol was the highest. This result showed that 75% ethanol in the gastric mucosa of the rats caused serious damage. Compared with that of the model group, the ulcer index of each administration group decreased to varying degrees. The SGD (19.80 g/kg) group and SGD (9.90 g/kg) group elicited an obvious lowering of the gastric ulcer index scores (*P* < 0.01); the ulcer inhibition rate was as high as 99.630% and 98.441%, respectively, and the protective effect was the best. These findings signify the efficacy of SGD in lowering the severity of gastric damage invoked by ethanol administration.

### 3.2. Histological Findings

**Figure 3:**
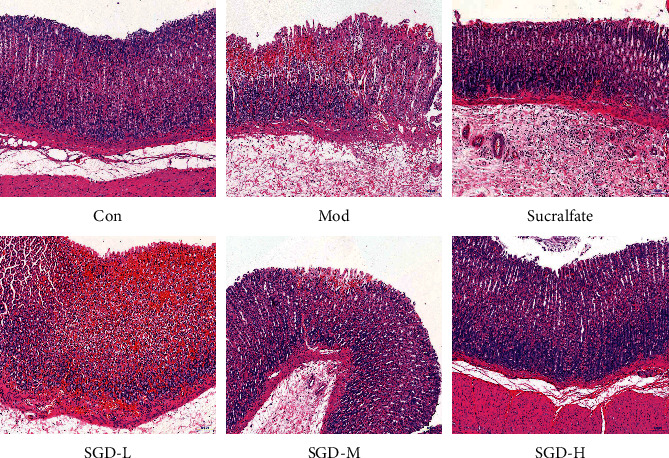
Effect of SGD on the pathological morphology of gastric mucosa in model rats by H&E staining (×100) (*n* = 8).

As shown in Figure 3, the results of H&E staining showed that the epithelial cells of the gastric mucosa were intact, and the inflammatory cells were normal, without infiltration, congestion, or edema. Compared with the control group, the full-thickness injury of the gastric mucosa in the model group was more obvious, the mucosal epithelium and the lamina propria were necrotic, the mucosal epithelium was missing, the morphology and structure of the tubular gastric glands in the lamina propria were blurred, a large number of parietal cells and principal cells were necrotic, and the cell nucleus was lysed. The cytoplasm was dissolved or absent, indicating that 75% ethanol caused serious damage to the gastric mucosa of the rats. Compared with that in the model group, the degree of gastric tissue lesions in the SGD (4.95 g/kg) group was not significantly reduced. In the SGD (9.90 g/kg) group, the superficial mucosa of the gastric tissue was slightly injured. The submucosa, muscle layer, and adventitia were relatively complete, with obvious stratification. The submucosa showed different degrees of inflammatory cell infiltration. The degree of lesions in the sucralfate group was similar to that in the SGD (9.90 g/kg) group. The degree of lesions in the SGD (19.80 g/kg) group was the smallest; the structure of the mucous layer, submucosa, muscle layer, and adventitia of the gastric tissue was more intact and stratified obviously; and there was no obvious edema in the submucosa and slight infiltration of inflammatory cells.

### 3.3. Effect of SGD on the Production of Inflammatory Markers

**Figure 4:**
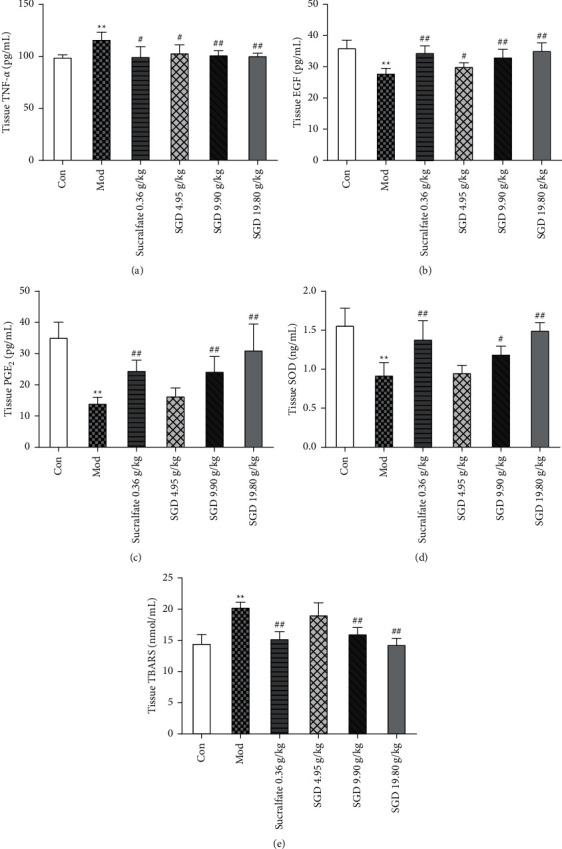
Effect of SGD on the levels of (a) TNF-*α*, (b) EGF, (c) PGE_2_, (d) SOD, and (e) TBARS in gastric tissue of model rats. Values are mean ± SD from 8 samples. Compared with the control group, ^*∗*^*P* < 0.05, ^*∗∗*^*P* < 0.01; compared with the model group, the remaining groups are ^#^*P* < 0.05, ^##^*P* < 0.01.

As shown in Figure 4(a), compared with that of the control group, the level of TNF-*α* in the gastric tissue of the ethanol-induced GU group increased (1.17-fold change), which triggered an inflammatory response. However, compared with those in the model group, the levels of TNF-*α* in the sucralfate group and the SGD (4.95 g/kg) group were reduced (85.83% and 88.87% of the model group, respectively, *P* < 0.05). The levels of TNF-*α* in the SGD (9.90 g/kg) group and SGD (19.8 g/kg) group were significantly reduced (87.17% and 86.50% of the model group, respectively, *P* < 0.01). These findings indicate that SGD can reduce the expression level of TNF-*α* in the gastric tissue of rats with GU, reduce inflammation, and protect the gastric mucosa.

### 3.4. Effect of SGD on the Release of Protective Mediators in the Gastric Mucosa

Figures 4(b) and 4(c) show the effect of SGD on the levels of EGF and PGE_2_ in the gastric tissue of rats with GU. Statistical analysis showed that compared with those in the control group, the levels of EGF and PGE_2_ in the gastric tissue of the ethanol-induced GU model group were significantly reduced (1.28-fold change for EGF and 2.49-fold change for PGE_2_, respectively, *P* < 0.01). Compared with those of the model group, the EGF levels of the sucralfate group, the SGD (9.90 g/kg) group, and the SGD (19.80 g/kg) group were significantly increased (123.53%, 118.45%, and 125.90% of the model group, respectively, *P* < 0.01). Similarly, the levels of PGE_2_ in the sucralfate group, SGD (9.90 g/kg) group, and SGD (19.80 g/kg) group were significantly higher than those in the model group (174.15%, 172.40%, and 220.86% of the model group, respectively, *P* < 0.01). These findings suggest that SGD can increase the expression of EGF and PGE_2_ in the gastric tissue of rats with GU and improve the protective function of the gastric mucosal barrier.

### 3.5. Effect of SGD on the Production of Oxidative Stress Markers

Ethanol-induced gastric injury is mediated by increased production of reactive oxygen species (ROS). The defense mechanism against ROS is mainly reflected in the increase in endogenous antioxidant enzyme levels and the inhibition of lipid peroxidation. As shown in Figure 4(d), in terms of antioxidant enzymes, the level of SOD in the gastric tissue of the ethanol-induced GU model group was significantly lower than that of the control group (1.69-fold change). Compared with that in the model group, the level of SOD in the sucralfate group and the SGD (19.80 g/kg) group increased significantly (149.78% and 162.13% of that in the model group, respectively, *P* < 0.01). The level of SOD in the SGD (9.90 g/kg) group increased (129.16% of that in the model group, *P* < 0.05). As shown in Figure 4(e), in terms of lipid peroxidation, the level of thiobarbituric acid reactive substances (TBARS) in the gastric tissue of rats in the ethanol-induced GU model group was significantly higher than that of rats in the control group (1.37-fold change, *P* < 0.01). Compared with that in the model group, the level of TBARS in the sucralfate group, SGD (9.90 g/kg) group, and SGD (19.80 g/kg) group decreased significantly (77.10%, 78.66%, and 71.83% of that in the model group, respectively, *P* < 0.01). These findings suggest that SGD can effectively increase the expression level of SOD in rat gastric tissue, reduce the expression level of TBARS in rat gastric tissue, and reduce oxidative stress injury, thus protecting the gastric mucosa.

### 3.6. Apoptosis Indexes

**Figure 5:**
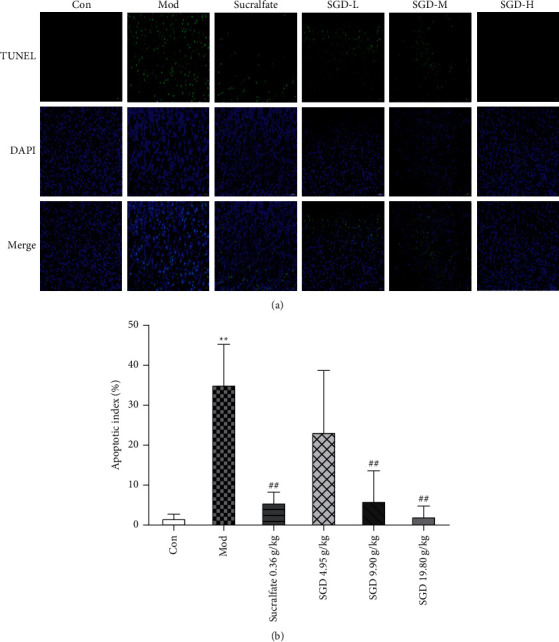
(a) Effect of SGD on apoptosis of gastric mucosal cells in model rats (*n* = 8). (b) Percentage of apoptotic cells in different experimental groups. Compared with the control group, ^*∗*^*P* < 0.05, ^*∗∗*^*P* < 0.01; compared with the model group, the remaining groups are ^#^*P* < 0.05, ^##^*P* < 0.01.

Under a fluorescence microscope (400x), the apoptotic nucleus showed green fluorescence. As shown in Figure 5, the percentage of apoptosis in the control group was relatively low, only 1.713 ± 1.012%, while the percentage of apoptosis in the gastric tissue of rats in the ethanol-induced GU model group was significantly increased (20.53-fold change, *P* < 0.01). After prophylactic administration, the percentage of apoptosis in gastric tissue of rats in each treatment group was lower than that in the model group. The percentage of apoptosis in the gastric tissue in the sucralfate group, SGD (9.90 g/kg) group, and SGD (19.80 g/kg) group was significantly lower than that in the model group (16.02%, 17.23%, and 6.28% of that in the model group, respectively, *P* < 0.01). In the SGD (4.95 g/kg) group, the percentage of apoptosis in gastric tissue decreased slightly, but there was no statistical significance (*P* > 0.05). The results showed that SGD could reduce apoptosis in the gastric tissue of rats with GU induced by ethanol.

### 3.7. Immunohistochemistry of the Caspase-3 and Bcl-2 Proteins

**Figure 6:**
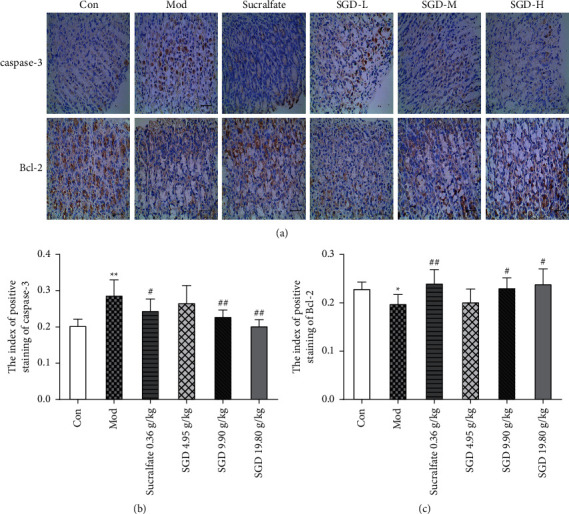
Effect of SGD on the protein expression of caspase-3 and Bcl-2 in gastric tissue of model rats (*n* = 6). (a) Representative graphs of each group. (b) Expression of caspase-3 protein in different experimental groups. (c) Expression of Bcl-2 protein in different experimental groups. Compared with the control group, ^*∗*^*P* < 0.05, ^*∗∗*^*P* < 0.01; compared with the model group, the rest of the group, ^#^*P* < 0.05, ^##^*P* < 0.01.

Immunohistochemical analyses were conducted to detect the expression of caspase-3 and Bcl-2 in gastric tissue (see Figure 6(a)). Immunohistochemistry of gastric slices showed that the expression level of Bcl-2 in the control group was stronger and the expression level of caspase-3 was weaker. In contrast, the expression level of caspase-3 increased and the expression level of Bcl-2 decreased in the ethanol-induced GU model group (see Figures 6(b) and 6(c)). The average optical densities of caspase-3 and Bcl-2 in gastric slices of rats in the control group were 0.204 ± 0.018 and 0.230 ± 0.013, respectively. After ethanol-induced GU, the average optical density of caspase-3 increased (1.41-fold change, *P* < 0.01), and the average optical density of Bcl-2 decreased (86.55% of the control group, *P* < 0.05). Compared with the model group, each administration group had different degrees of improvement, but the SGD (4.95 g/kg) group did not reach the standard of statistical significance. The average optical density of caspase-3 in the gastric tissue of the sucralfate group, SGD (9.90 g/kg) group, and SGD (19.80 g/kg) group was significantly lower than that in the model group (85.29%, 79.52%, and 70.20% of the model group, respectively, *P* < 0.05 or *P* < 0.01). The average optical density of Bcl-2 in the gastric tissue of the sucralfate group, SGD (9.90 g/kg) group, and SGD (19.80 g/kg) group increased significantly (121.26%, 116.44%, and 118.87% of that in the model group, respectively, *P* < 0.05 or *P* < 0.01). These results indicate that SGD treatment increases the expression of the Bcl-2 protein in gastric tissue after ethanol-induced GU and reduces the expression of the caspase-3 protein.

### 3.8. Network Pharmacology Analysis

#### 3.8.1. SGD Active Ingredient Screening and Target Acquisition

**Figure 7:**
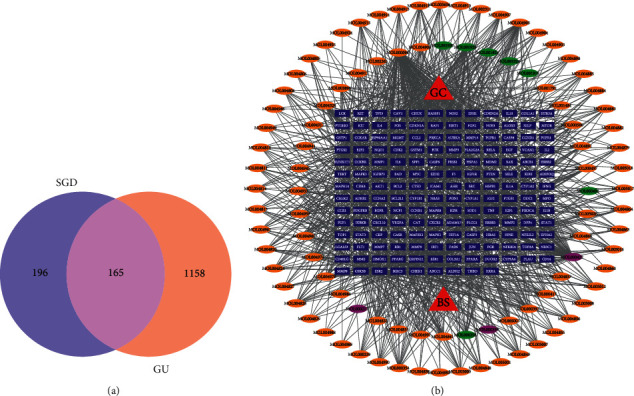
Venn diagram of SGD targets and “component-target” network diagram. (a) Venn diagram of SGD target point. (b) “Component-target” network diagram of SGD. The red node is medicinal materials, the orange node is the active ingredient of GC, the green node is the active ingredient of BS, the pink node is the common ingredient of BS and GC, and the blue node is the target protein.

A total of 365 chemical ingredients of BS and GC were retrieved from TCMSP. A total of 105 active compounds were screened out following the OB ≥ 30% and DL ≥ 0.18 criteria. After deduplication, 102 active ingredients of SGD were obtained. A total of 232 potential human targets were found through the TCMSP database, and 165 potential human targets were found through the Swiss Target Prediction database. After the deletion of duplicate targets, a total of 361 targets were obtained (see Figure 7(a)).

#### 3.8.2. Acquisition of GU Disease Targets and Construction of a Component-Target Network

The structures of 1323 GU-related targets were retrieved from four databases, including the DrugBank database. Then, 361 targets of SGD and 1323 related targets for GU were imported into Draw Venn Diagram (http://bioinformatics.psb.ugent.be/webtools/Venn/), yielding 165 common targets, which are the key targets of SGD treatment of GU, as shown in Figure 7(a). As shown in Figure 7(b), Cytoscape 3.7.2 was used to construct the drug-active ingredient-target network. The relationship between the components and the target was visible, and the network topology was analyzed. The network consists of 263 nodes (2 drugs, 96 active ingredients, and 165 targets) and 1208 edges. For the 6 active ingredients, no relevant targets were found, so the constructed network diagram showed only 96 active ingredients. In this network, multiple active ingredients work together on the same target protein, and a single active ingredient is associated with multiple target proteins, reflecting the multicomponent and multitarget interactions of traditional Chinese medicine compounds.

#### 3.8.3. Construction of the PPI Network and Screening of Key Targets

**Figure 8:**
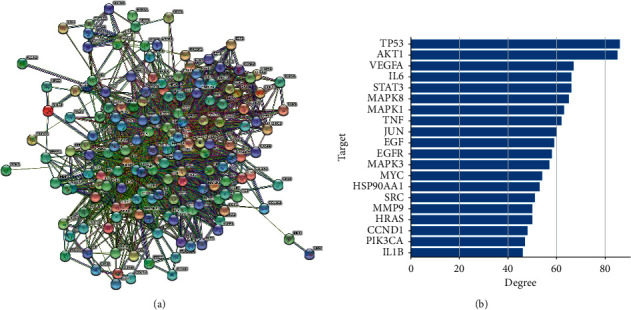
Protein-protein interaction network diagram of SGD active ingredients against gastric ulcer targets. (a) PPI network diagram. (b) Top 20 key targets.

We imported 165 key targets into the STRING database to obtain the PPI network diagram, as shown in Figure 8(a). The obtained results were imported into Cytoscape 3.7.2, and the network topology was analyzed. The network includes 163 nodes and 1782 edges. After network topology analysis, the top 20 key targets were ranked, as shown in Figure 8(b). These targets play a key role in the PPI protein interaction network and may occupy an important position in the treatment of GU. Using the PPI network, we explored the relationship between different target genes and laid the foundation for subsequent pathway analysis.

#### 3.8.4. Enrichment Analysis

**Figure 9:**
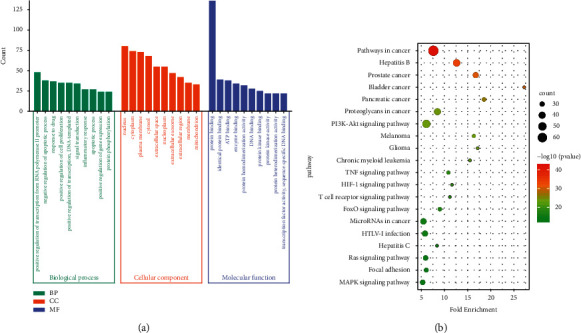
GO biological function and pathway enrichment analysis diagram of key targets. (a) Top ten GO biological functions. (b) Top 20 pathways.

Disease occurrence and drug treatment are dynamic processes that interact with multiple factors, including DNA damage, gene mutation, cell cycle behavior, population dynamics, inflammation, and metabolic immune balance. To explore the dynamic processes associated with the anti-GU effect of SGD, the key targets were imported into the DAVID 6.8 online analysis platform (https://david.ncifcrf.gov/), and the potential key targets of the active ingredients of SGD were analyzed by the GO functional enrichment analysis. The identifier was “OFFICIAL GENE SYMBOL,” the species was “Homo sapiens,” and the screening condition was a *P* value < 0.01. The results of GO enrichment analysis included different biological processes (BPs), cell components (CCs), and molecular functions (MFs). After analysis, the results were sorted by count value, and the top 10 biological functions of the three modules were obtained; the results are shown in Figure 9(a). BP analysis involves positive regulation of transcription from the RNA polymerase II promoter, negative regulation of apoptosis, response to drug, positive regulation of cell proliferation, inflammatory response, apoptosis process, etc. CC analysis involves the nucleus, cytoplasm, plasma membrane, cytosol, extracellular space, and so on. MF analysis involves protein binding, identical protein binding, ATP binding, enzyme binding, protein homodimerization activity, etc. The findings suggest that SGD plays a role in the process of resisting GU by participating in the regulation of a variety of biological processes.

The key targets were imported into the DAVID 6.8 online analysis platform (https://david.ncifcrf.gov/), and KEGG pathway enrichment analysis was performed on the potential key targets of the active ingredients of SGD, with the identifier “OFFICIAL GENE SYMBOL,” the species “Homo sapiens,” and the selection condition *P* value < 0.01. Sorted by the count value, after analysis, the top 20 pathways are shown in Figure 9(b). Involvement of the PI3K-Akt signaling pathway, Ras signaling pathway, MAPK signaling pathway, FoxO signaling pathway, TNF signaling pathway, etc., suggests that SGD exerts an anti-GU effect by acting on multiple pathways.

## 4. Discussion

GU is one of the most common diseases affecting the digestive system, and it affects millions of people around the world. GU is mainly caused by the imbalance of aggressive and defensive factors in the stomach [37]. Ethanol is one of the main causes of GU-related injury. After ethanol-induced GU formation, gastric tissue shows mucosal edema, mucosal epithelium loss, necrosis, apoptosis, and hemorrhage [38]. The pathogenic factors of ethanol-induced GU are complex and closely related to changes in pro-inflammatory cytokines, gastric mucosal protective factors, and apoptotic proteins. GU is a common gastrointestinal dysfunction disease. The clinical use of drugs for prevention and treatment differs according to the varying pathogenic factors observed in GU. Generally, drugs can be subdivided into acid secretion drugs, mucosal resistance-enhancing drugs and inhibitors, *Helicobacter pylori*-targeting drugs, etc. Among them, the most commonly used are proton pump inhibitor drugs. However, long-term use of these drugs may cause adverse reactions, such as nausea and vomiting. Therefore, an increasing number of scholars are focusing on traditional Chinese medicine compounds as alternative medicines to prevent GU.

SGD, as one of the 100 classic prescriptions in “The Catalogue of Ancient Classical Prescriptions (First Batch),” has simplified prescriptions, clear compatibility, accurate curative effects, and rich clinical use records, and it has extremely important research and development value. In this study, 75% ethanol was used to establish a rat GU model to verify the anti-GU effect of SGD from four perspectives: anti-inflammatory effects, gastric mucosal protection, antioxidative effects, and antiapoptosis effects. Network pharmacology research methods were used to study the potential mechanism underlying the anti-GU effect of SGD and explain the scientific connotation of SGD's anti-GU effects.

Inflammation is an important aspect of the pathogenesis of GU, and a variety of inflammatory cytokines are involved. TNF-*α* is particularly important for mediating the occurrence of GU. TNF-*α* is mainly an effector molecule with a wide range of biological activities that is secreted by mast cells, endothelial cells, and mononuclear phagocytes. It effectively stimulates neutrophil infiltration. Factors can activate neutrophils to accumulate in large numbers around the ulcer, hinder the blood microcirculation around the ulcer, cause gastric microcirculation disorders, and induce the formation of ulcers. In this study, after normal rats were given 75% ethanol by gavage, the level of TNF-*α* in the gastric tissue of the rats increased, and the administration of SGD could reduce the expression level of TNF-*α* in the gastric tissue of rats with GU and reduce inflammation. Our research results are consistent with previous studies [39], which indicate that the anti-inflammatory activity of SGD endows it with a gastroprotective effect to a certain extent.

EGF and PGE_2_ are important defense and repair factors of the gastric mucosa. EGF can inhibit the secretion of gastric acid and pepsin, reduce its damage to the mucosa, increase the synthesis and secretion of gastric mucosa and glycoproteins, mediate its nutritional and protective effects on the gastric mucosa, and maintain the integrity of the gastric mucosa. PGE_2_ can increase local gastric mucosal blood flow, stimulate gastric mucosal basal cells to migrate to the surface, promote mucosal repair, maintain mucosal integrity, and enhance mucosal defense functions. In this study, the levels of EGF and PGE_2_ in the gastric tissue of rats in the ethanol-induced GU group were significantly reduced, while the levels of EGF and PGE_2_ were significantly increased after the administration of SGD. Our results are consistent with those of many previous studies. These findings indicate that SGD can promote gastric mucosal synthesis, secretion, and release of PGE_2_ and increase endogenous EGF levels, thereby promoting gastric mucosal damage repair and enhancing gastric mucosal barrier defense function.

Oxidative stress refers to the imbalance between the production and removal of oxygen free radicals in organisms or cells, which induces the accumulation of reactive oxygen species (ROS) in the body or cells and then causes oxidation. SOD, an important intracellular antioxidant enzyme, constitutes the first line of defense against ROS. It catalyzes the conversion of superoxide free radicals into more stable hydrogen peroxide, which is then decomposed into completely harmless water by catalase in the body, protecting cells from the toxic damage of ROS [40]. Excessive active oxygen can cause lipids, proteins, nucleic acids, and other cellular components to undergo lipid peroxidation, thereby inducing dysfunction, increasing the level of TBARS [41], and severely damaging the surface of the gastric tissue. This process plays a key role in the pathogenesis of mucosal damage. In this study, after normal rats were given 75% ethanol by gavage, the SOD level in the rat gastric tissue was significantly reduced, and the TBARS level was significantly increased. SGD administration can effectively increase the SOD expression level in rat gastric tissue and reduce the rat TBARS expression level in stomach tissues. These findings indicate that SGD can reduce oxidative stress damage, thereby protecting the gastric mucosa.

The occurrence of apoptosis of gastric mucosal epithelial cells is closely related to the occurrence of ulcers [42]. Gastric mucosal epithelial cells have the ability to continuously renew; that is, they have a certain repair function after injury. Under normal circumstances, gastric mucosal epithelial cells are in a balance between apoptosis and proliferation. However, when the gastric mucosa suffers severe damage, it will destroy the structure and function of gastric epithelial cells, break the balance of their proliferation and apoptosis, and cause GU. Bcl-2 is closely related to apoptosis. It was the first protein found to inhibit cell apoptosis [43], and it plays an important role in controlling cell survival and death. Bcl-2 primarily maintains the dynamic balance of Ca^2+^ and antagonizes proapoptotic proteins. The expression and stabilization of mitochondrial membranes inhibit cell apoptosis. Caspase-3 is an indispensable protease in the apoptotic protease cascade, and its activation can eventually cause cell death [30]. In this study, the percentage of apoptosis in the gastric tissue of rats in the ethanol-induced GU model group was significantly increased, the expression level of Bcl-2 decreased, and the expression level of caspase-3 increased. After the administration of SGD, the percentage of apoptosis in rat gastric tissues decreased to varying degrees, the expression level of Bcl-2 increased, and the expression level of caspase-3 decreased significantly. SGD reduced ethanol-induced apoptosis of gastric tissue in rats with GU.

Network pharmacology is a form of big data research and differs from traditional single-component, single-target thinking. Its multicomponent, multitarget, and multichannel network construction approach is more suitable for the complex characteristics of traditional Chinese medicine compounds. Network pharmacology is based on omics and integrated with systems biology. It provides a large amount of information on compounds, targets, diseases, etc., related to the pharmacological effects of single and compound Chinese medicines. This approach can be used to analyze the mechanism of action of traditional Chinese medicines from the perspective of modern pharmacology. This type of analysis provides a powerful tool for exploring the mechanisms of action of traditional Chinese medicines and developing active ingredients of traditional Chinese medicines [44, 45]. Network pharmacology is also visually presented through the network structure diagram of “Traditional Chinese Medicine-Ingredients-Targets–Diseases,” which can effectively reveal the key nodes of traditional Chinese medicine compounds [46–49], predict the potential mechanisms of traditional Chinese medicines, and discover innovative drugs [50].

The PI3K-Akt pathway is widely present in cells. It is a signal transduction pathway that participates in the regulation of cell growth, proliferation, and differentiation. It is also the main pathway through which a variety of anti-ulcer drugs exert their effects [51]. Existing studies have shown that the activation of the PI3K-Akt signaling pathway and the repair of gastric mucosal damage have a very important role; this process can accelerate cell metabolism, inhibit cell apoptosis, and play an important role in cell survival, cell infiltration, metastasis, and other biological activities [52].

The results of this study directly prove that SGD can significantly inhibit the formation of GU and protect against gastric mucosal damage in rats with GU induced by ethanol. The mechanism involves multiple pathways, which are closely related to reducing inflammation, improving the antioxidant performance of gastric tissue, improving the protective function of the gastric mucosal barrier, and inhibiting cell apoptosis. The anti-GU effect of SGD may be related to the activation of gastric mucosal cells. This effect is related to multiple signaling pathways, such as PI3K-Akt.

## 5. Conclusions

The above results show that SGD has a protective effect on the gastric mucosa of rats, has a protective effect on alcohol-induced GU-related injury, and can promote ulcer healing. The protective effect of SGD is at least partially explained by anti-inflammatory effects, EGF production, PGE_2_ production, antioxidative stress effects, and antiapoptotic effects. In addition, we screened the potential mechanism underlying the anti-GU effect of SGD through network pharmacology for the first time and found that the PI3K-Akt signaling pathway, Ras signaling pathway, MAPK signaling pathway, and other signaling pathways play an important role in the anti-GU effect of SGD. These findings show that SGD plays a role in anti-GU effects by participating in the regulation of multiple biological processes and acting on multiple pathways, which reflects the multicomponent, multitarget, and multichannel regulation that is characteristic of traditional Chinese medicine compounds. The potential mechanism of SGD's ability to protect against GU needs to be further verified, and a clearer functional mechanism and kinetic basis are needed to provide more scientific and reliable experimental data support for the clinical application of SGD.

## Data Availability

The data used to support the findings of this study are included within the article.

## Abbreviations

GU: Gastric ulcer
SGD: Shaoyao-Gancao decoction
BS: Baishao
ZGC: Zhi Gan Cao
TCM: Traditional Chinese medicine
UI: Ulcer index
TNF-*α*: Tumor necrosis factor-*α*
EGF: Epidermal growth factor
PGE_2_: Prostaglandin E_2_
SOD: Superoxide dismutase
TBARS: Thiobarbituric acid reactive substances
SPSS: Statistical Package for the Social Sciences
TCMSP: Traditional Chinese Medicine Systems Pharmacology Database and Analysis Platform
OMIM: Online Mendelian Inheritance in Man
ADME: Absorption, distribution, metabolism, and excretion
OB: Oral bioavailability
DL: Drug likeness
STRING: Search Tool for the Retrieval of Interacting
PPI: Protein-protein interaction
GO: Gene ontology
KEGG: Kyoto Encyclopedia of Genes and Genomes
CC: Cellular components
MF: Molecular functions
BP: Biological processes.

## Supplementary Materials

Supplementary Materials Table S1: active ingredients in SGD. Fig S1: content of five ingredients in SGD. Table S2: content of five ingredients in SGD.
