# Supplementary material for: Protective Effect and Potential Mechanism of the Traditional Chinese Medicine Shaoyao-Gancao Decoction on Ethanol-Induced Gastric Ulcers in Rats
Source: Evid Based Complement Alternat Med. 2022 Apr 11;2022:3069089. doi: 10.1155/2022/3069089 (PMC9017495; doi:10.1155/2022/3069089)
Supplement: Supplementary Materials — Table S1: active ingredients in SGD. Fig S1: content of five ingredients in SGD. Table S2: content of five ingredients in SGD. [file 3069089.f1.docx]

**Supplementary material**

Table S1 Active ingredients in SGD

| Number | Mol ID | Molecule Name | OB (%) | DL | Drug |
| --- | --- | --- | --- | --- | --- |
| 1 | MOL001910 | 11alpha,12alpha-epoxy-3beta-23-dihydroxy-30-norolean-20-en-28,12beta-olide | 64.77 | 0.38 | BS |
| 2 | MOL001918 | paeoniflorgenone | 87.59 | 0.37 | BS |
| 3 | MOL001919 | (3S,5R,8R,9R,10S,14S)-3,17-dihydroxy-4,4,8,10,14-pentamethyl-2,3,5,6,7,9-hexahydro-1H-cyclopenta[a]phenanthrene-15,16-dione | 43.56 | 0.53 | BS |
| 4 | MOL001921 | Lactiflorin | 49.12 | 0.8 | BS |
| 5 | MOL001924 | paeoniflorin | 53.87 | 0.79 | BS |
| 6 | MOL001925 | paeoniflorin_qt | 68.18 | 0.4 | BS |
| 7 | MOL001928 | albiflorin_qt | 66.64 | 0.33 | BS |
| 8 | MOL001930 | benzoyl paeoniflorin | 31.27 | 0.75 | BS |
| 9 | MOL000358 | beta-sitosterol | 36.91 | 0.75 | BS |
| 10 | MOL000211 | Mairin | 55.38 | 0.78 | BS+GC |
| 11 | MOL000359 | sitosterol | 36.91 | 0.75 | BS+GC |
| 12 | MOL000422 | kaempferol | 41.88 | 0.24 | BS+GC |
| 13 | MOL000492 | (+)-catechin | 54.83 | 0.24 | BS |
| 14 | MOL001484 | Inermine | 75.18 | 0.54 | GC |
| 15 | MOL001792 | DFV | 32.76 | 0.18 | GC |
| 16 | MOL002311 | Glycyrol | 90.78 | 0.67 | GC |
| 17 | MOL000239 | Jaranol | 50.83 | 0.29 | GC |
| 18 | MOL002565 | Medicarpin | 49.22 | 0.34 | GC |
| 19 | MOL000354 | isorhamnetin | 49.6 | 0.31 | GC |
| 20 | MOL003656 | Lupiwighteone | 51.64 | 0.37 | GC |
| 21 | MOL003896 | 7-Methoxy-2-methyl isoflavone | 42.56 | 0.2 | GC |
| 22 | MOL000392 | formononetin | 69.67 | 0.21 | GC |
| 23 | MOL000417 | Calycosin | 47.75 | 0.24 | GC |
| 24 | MOL004328 | naringenin | 59.29 | 0.21 | GC |
| 25 | MOL004805 | (2S)-2-[4-hydroxy-3-(3-methylbut-2-enyl)phenyl]-8,8-dimethyl-2,3-dihydropyrano[2,3-f]chromen-4-one | 31.79 | 0.72 | GC |
| 26 | MOL004806 | euchrenone | 30.29 | 0.57 | GC |
| 27 | MOL004808 | glyasperin B | 65.22 | 0.44 | GC |
| 28 | MOL004810 | glyasperin F | 75.84 | 0.54 | GC |
| 29 | MOL004811 | Glyasperin C | 45.56 | 0.4 | GC |
| 30 | MOL004814 | Isotrifoliol | 31.94 | 0.42 | GC |
| 31 | MOL004815 | (E)-1-(2,4-dihydroxyphenyl)-3-(2,2-dimethylchromen-6-yl)prop-2-en-1-one | 39.62 | 0.35 | GC |
| 32 | MOL004820 | kanzonols W | 50.48 | 0.52 | GC |
| 33 | MOL004824 | (2S)-6-(2,4-dihydroxyphenyl)-2-(2-hydroxypropan-2-yl)-4-methoxy-2,3-dihydrofuro[3,2-g]chromen-7-one | 60.25 | 0.63 | GC |
| 34 | MOL004827 | Semilicoisoflavone B | 48.78 | 0.55 | GC |
| 35 | MOL004828 | Glepidotin A | 44.72 | 0.35 | GC |
| 36 | MOL004829 | Glepidotin B | 64.46 | 0.34 | GC |
| 37 | MOL004833 | Phaseolinisoflavan | 32.01 | 0.45 | GC |
| 38 | MOL004835 | Glypallichalcone | 61.6 | 0.19 | GC |
| 39 | MOL004838 | 8-(6-hydroxy-2-benzofuranyl)-2,2-dimethyl-5-chromenol | 58.44 | 0.38 | GC |
| 40 | MOL004841 | Licochalcone B | 76.76 | 0.19 | GC |
| 41 | MOL004848 | licochalcone G | 49.25 | 0.32 | GC |
| 42 | MOL004849 | 3-(2,4-dihydroxyphenyl)-8-(1,1-dimethylprop-2-enyl)-7-hydroxy-5-methoxy-coumarin | 59.62 | 0.43 | GC |
| 43 | MOL004855 | Licoricone | 63.58 | 0.47 | GC |
| 44 | MOL004856 | Gancaonin A | 51.08 | 0.4 | GC |
| 45 | MOL004857 | Gancaonin B | 48.79 | 0.45 | GC |
| 46 | MOL004860 | licorice glycoside E | 32.89 | 0.27 | GC |
| 47 | MOL004863 | 3-(3,4-dihydroxyphenyl)-5,7-dihydroxy-8-(3-methylbut-2-enyl)chromone | 66.37 | 0.41 | GC |
| 48 | MOL004864 | 5,7-dihydroxy-3-(4-methoxyphenyl)-8-(3-methylbut-2-enyl)chromone | 30.49 | 0.41 | GC |
| 49 | MOL004866 | 2-(3,4-dihydroxyphenyl)-5,7-dihydroxy-6-(3-methylbut-2-enyl)chromone | 44.15 | 0.41 | GC |
| 50 | MOL004879 | Glycyrin | 52.61 | 0.47 | GC |
| 51 | MOL004882 | Licocoumarone | 33.21 | 0.36 | GC |
| 52 | MOL004883 | Licoisoflavone | 41.61 | 0.42 | GC |
| 53 | MOL004884 | Licoisoflavone B | 38.93 | 0.55 | GC |
| 54 | MOL004885 | licoisoflavanone | 52.47 | 0.54 | GC |
| 55 | MOL004891 | shinpterocarpin | 80.3 | 0.73 | GC |
| 56 | MOL004898 | (E)-3-[3,4-dihydroxy-5-(3-methylbut-2-enyl)phenyl]-1-(2,4-dihydroxyphenyl)prop-2-en-1-one | 46.27 | 0.31 | GC |
| 57 | MOL004903 | liquiritin | 65.69 | 0.74 | GC |
| 58 | MOL004904 | licopyranocoumarin | 80.36 | 0.65 | GC |
| 59 | MOL004905 | 3,22-Dihydroxy-11-oxo-delta(12)-oleanene-27-alpha-methoxycarbonyl-29-oic acid | 34.32 | 0.55 | GC |
| 60 | MOL004907 | Glyzaglabrin | 61.07 | 0.35 | GC |
| 61 | MOL004908 | Glabridin | 53.25 | 0.47 | GC |
| 62 | MOL004910 | Glabranin | 52.9 | 0.31 | GC |
| 63 | MOL004911 | Glabrene | 46.27 | 0.44 | GC |
| 64 | MOL004912 | Glabrone | 52.51 | 0.5 | GC |
| 65 | MOL004913 | 1,3-dihydroxy-9-methoxy-6-benzofurano[3,2-c]chromenone | 48.14 | 0.43 | GC |
| 66 | MOL004914 | 1,3-dihydroxy-8,9-dimethoxy-6-benzofurano[3,2-c]chromenone | 62.9 | 0.53 | GC |
| 67 | MOL004915 | Eurycarpin A | 43.28 | 0.37 | GC |
| 68 | MOL004917 | glycyroside | 37.25 | 0.79 | GC |
| 69 | MOL004924 | (-)-Medicocarpin | 40.99 | 0.95 | GC |
| 70 | MOL004935 | Sigmoidin-B | 34.88 | 0.41 | GC |
| 71 | MOL004941 | (2R)-7-hydroxy-2-(4-hydroxyphenyl)chroman-4-one | 71.12 | 0.18 | GC |
| 72 | MOL004945 | (2S)-7-hydroxy-2-(4-hydroxyphenyl)-8-(3-methylbut-2-enyl)chroman-4-one | 36.57 | 0.32 | GC |
| 73 | MOL004948 | Isoglycyrol | 44.7 | 0.84 | GC |
| 74 | MOL004949 | Isolicoflavonol | 45.17 | 0.42 | GC |
| 75 | MOL004957 | HMO | 38.37 | 0.21 | GC |
| 76 | MOL004959 | 1-Methoxyphaseollidin | 69.98 | 0.64 | GC |
| 77 | MOL004961 | Quercetin der. | 46.45 | 0.33 | GC |
| 78 | MOL004966 | 3'-Hydroxy-4'-O-Methylglabridin | 43.71 | 0.57 | GC |
| 79 | MOL000497 | licochalcone a | 40.79 | 0.29 | GC |
| 80 | MOL004974 | 3'-Methoxyglabridin | 46.16 | 0.57 | GC |
| 81 | MOL004978 | 2-[(3R)-8,8-dimethyl-3,4-dihydro-2H-pyrano[6,5-f]chromen-3-yl]-5-methoxyphenol | 36.21 | 0.52 | GC |
| 82 | MOL004980 | Inflacoumarin A | 39.71 | 0.33 | GC |
| 83 | MOL004985 | icos-5-enoic acid | 30.7 | 0.2 | GC |
| 84 | MOL004988 | Kanzonol F | 32.47 | 0.89 | GC |
| 85 | MOL004989 | 6-prenylated eriodictyol | 39.22 | 0.41 | GC |
| 86 | MOL004990 | 7,2',4'-trihydroxy－5-methoxy-3－arylcoumarin | 83.71 | 0.27 | GC |
| 87 | MOL004991 | 7-Acetoxy-2-methylisoflavone | 38.92 | 0.26 | GC |
| 88 | MOL004993 | 8-prenylated eriodictyol | 53.79 | 0.4 | GC |
| 89 | MOL004996 | gadelaidic acid | 30.7 | 0.2 | GC |
| 90 | MOL000500 | Vestitol | 74.66 | 0.21 | GC |
| 91 | MOL005000 | Gancaonin G | 60.44 | 0.39 | GC |
| 92 | MOL005001 | Gancaonin H | 50.1 | 0.78 | GC |
| 93 | MOL005003 | Licoagrocarpin | 58.81 | 0.58 | GC |
| 94 | MOL005007 | Glyasperins M | 72.67 | 0.59 | GC |
| 95 | MOL005008 | Glycyrrhiza flavonol A | 41.28 | 0.6 | GC |
| 96 | MOL005012 | Licoagroisoflavone | 57.28 | 0.49 | GC |
| 97 | MOL005013 | 18α-hydroxyglycyrrhetic acid | 41.16 | 0.71 | GC |
| 98 | MOL005016 | Odoratin | 49.95 | 0.3 | GC |
| 99 | MOL005017 | Phaseol | 78.77 | 0.58 | GC |
| 100 | MOL005018 | Xambioona | 54.85 | 0.87 | GC |
| 101 | MOL005020 | dehydroglyasperins C | 53.82 | 0.37 | GC |
| 102 | MOL000098 | quercetin | 46.43 | 0.28 | GC |


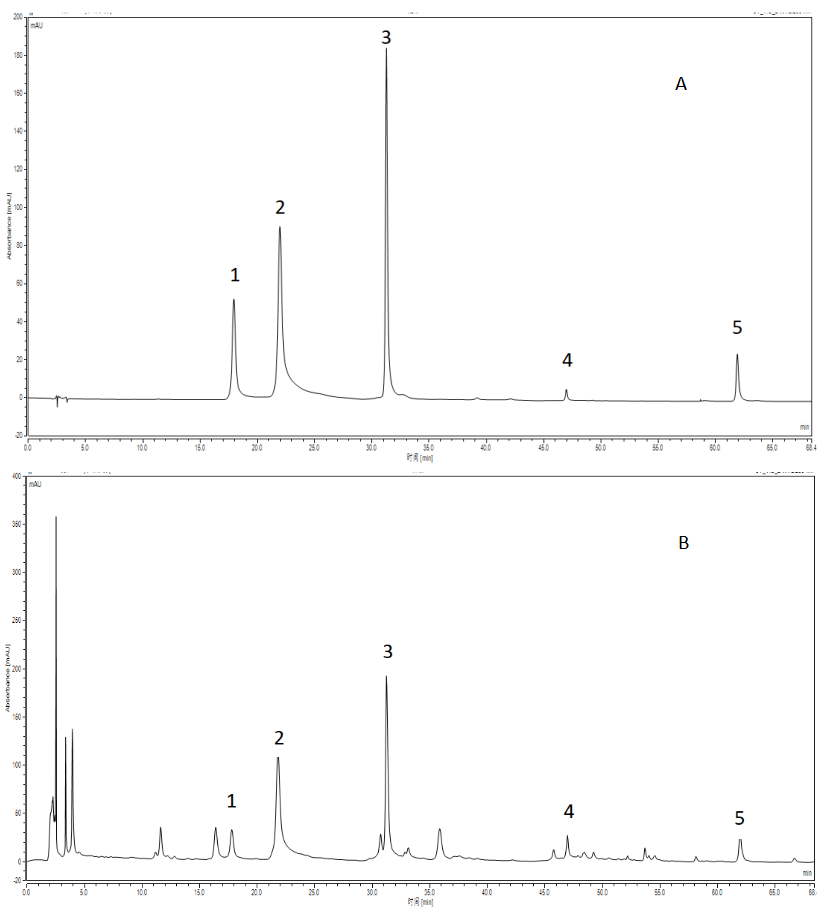


**Fig. S1** Content of five ingredients in SGD. (A) HPLC chromatograms of analytical standards (1-5). (B) HPLC chromatograms of SGD sample. Albiflorin(1), Paeoniflorin (2), Liquiritin (3), Isoliquiritin (4), glycyrrhizic acid (5).

**Table S****2** Content of five ingredients in SGD

| Ingredient | Content（mg/g） | Mean（mg/g） | RSD（%） |
| --- | --- | --- | --- |
| Albiflorin | 11.149 | 11.276 | 1.26 |
|  | 11.424 |  |  |
|  | 11.256 |  |  |
| Paeoniflorin | 34.105 | 34.481 | 2.23 |
|  | 35.100 |  |  |
|  | 34.241 |  |  |
| Liquiritin | 13.952 | 13.972 | 1.01 |
|  | 14.101 |  |  |
|  | 13.863 |  |  |
| Isoliquiritin | 1.538 | 1.542 | 0.73 |
|  | 1.555 |  |  |
|  | 1.533 |  |  |
| glycyrrhizic acid | 18.470 | 18.459 | 1.79 |
|  | 18.635 |  |  |
|  | 18.272 |  |  |
